# Supplementary material for: Understanding the determinants of treated bed net use in Ethiopia: A machine learning classification approach using PMA Ethiopia 2023 survey data
Source: PLoS One. 2025 Jul 7;20(7):e0327800. doi: 10.1371/journal.pone.0327800 (PMC12233236; doi:10.1371/journal.pone.0327800)
Supplement: Attachment — Submitted filename: Response to Reviewers.docx [file pone.0327800.s001.docx]

# **Response to Academic Editor**

Dear Rajib Chowdhury,

Thank you for your feedback, your professionalism, fast response, and for outlining the additional requirements for resubmission. I have carefully addressed each of the points raised, and I am pleased to submit the revised manuscript in compliance with PLOS ONE’s guidelines. Below is a summary of the revisions:

1. **Formatting and Style Requirements**:
   - The manuscript has been thoroughly revised to align with PLOS ONE’s style templates. This includes adjustments to file naming, section headings, reference formatting, and overall structure.
2. **Language Editing and Professional Copyediting**:
   - As suggested, the manuscript was professionally copyedited by with Grammarly to ensure clarity, proper grammar, spelling, and adherence to journal guidelines.
   - Attached as **Revised Manuscript with track changes** is a version of the manuscript with tracked changes highlighting all edits.
   - A clean, edited copy of the manuscript has been uploaded as the **new manuscript file**.
3. **Ethics Statement**:
   - The ethics statement has been moved exclusively to the Methods section, as instructed, and removed from any other part of the manuscript.

# **Response to Reviewers’ Comments**

Dear Reviewers,

Thank you for your constructive feedback and for the opportunity to improve my manuscript. I have carefully addressed all comments from both reviewers and revised the manuscript accordingly. Below is a point-by-point response to the reviewers’ concerns, along with details of the revisions made.

### **Reviewer #1**

1. **Define ITN (consistent) use**
   - **Revision:** I have clarified the definition of ITN use in the Methods section (under **Variables**):
     "Insecticide-treated net (ITN) use was evaluated at the household level through two criteria: (1) the confirmed presence of at least one ITN during physical inspection, and (2) verification that the net was both insecticide-treated and actively deployed (i.e., hung over a sleeping space) at the time of the survey. Households with nets that were untreated or not deployed were categorized as non-users."
2. **Multicollinearity check**
   - **Revision:** I confirmed in the Methods section (under **Preprocessing**) that multicollinearity was assessed using Variance Inflation Factor (VIF) analysis, with all values <10 (highest VIF = 0.72). We also clarified that variables like electricity access and media exposure were retained as distinct predictors due to their theoretical relevance beyond wealth quintile.
3. **Measurement level (household vs. individual)**
   - **Revision:** I emphasized in the Methods section that ITN use was measured at the household level. However, I added a limitation acknowledging that individual adherence (e.g., consistent use by all members) was not captured.
4. **Address sufficiency of ITNs**
   - **Revision:** I expanded the Discussion to include sufficiency metrics:
     "While our study focused on household-level ITN availability and deployment, future work should incorporate universal coverage thresholds (e.g., ≥1 ITN per 2 household members) to assess adequacy and its implications for malaria control." Because the available data doesn’t address it since the data is secondary not collected by me.
5. **Boldly discuss ITN use determinants**
   - **Revision:** I restructured the Results and Discussion sections to foreground determinants of ITN use (e.g., regional disparities, maternal education, and wealth quintiles) before elaborating on ML model performance.
6. **Revise Fig 2 (regional distribution)**
   - **Revision:** Figure 3 (regional distribution) has been updated to reflect Ethiopia’s current administrative regions (including Sidama and SWEP). Axis labels were adjusted to prevent overlap, and a clearer legend was added. It results in a change in some results and I make an update on them.

### **Reviewer #2**

1. **Clarify focus on ITN use vs. ownership**
   - **Revision:** I revised the title, abstract, and manuscript to consistently emphasize **ITN use** (not ownership). The outcome variable in the Methods section was redefined to explicitly measure use (deployment), not mere ownership. All instances of "ownership" were replaced with "use" where appropriate because the intent of the questionnaire was assessing their utilization of bed net (which is treated) correctly and I make define it correctly under Methods (Variables) section.
2. **Logistic regression assumptions**

Thank you for this critical observation. You are correct: logistic regression (LR) does not assume linearity between raw predictors and the outcome but instead models linearity in the log-odds space. Our original phrasing oversimplified this distinction, and we have revised the text to clarify that:

1. **LR requires manual specification of non-linear relationships** (e.g., polynomial terms, interaction effects) to approximate complex patterns.
2. **ML models inherently capture non-linearities and interactions** without explicit feature engineering, enabling them to model complex decision boundaries autonomously.

## **Revision:** I corrected the statement to be clear about logistic regression in the Discussion under Methodological Innovations and Policy Implications:

"*In contrast to logistic regression, which assumes linearity in the log-odds space and necessitates prior specification of non-linear terms (e.g., interactions or polynomial features), machine learning models autonomously adapt to non-linear and interactive relationships. This flexibility underscores their advantage in identifying complex determinants of ITN use, aligning with this study’s focus on uncovering patterns that may be overlooked by conventional parametric approaches*."

3. **Variable definitions and alignment with study focus**
   - **Revision:** The Variables section was revised to clarify that the outcome variable measures **ITN use** (deployment), not ownership and I have clarified the definition of ITN use in the Methods section (under **Variables**):
     "Insecticide-treated net (ITN) use was evaluated at the household level through two criteria: (1) the confirmed presence of at least one ITN during physical inspection, and (2) verification that the net was both insecticide-treated and actively deployed (i.e., hung over a sleeping space) at the time of the survey. Households with nets that were untreated or not deployed were categorized as non-users."
   - We also added a paragraph in the Discussion explicitly linking predictors (e.g., housing conditions, region) to behavioral compliance:
     "For instance, regional disparities in ITN deployment reflect differences in malaria risk perception, distribution efficiency, and cultural practices, which collectively influence use beyond mere ownership."

### **Additional Revisions**

- **Figures:** All figures (e.g., SHAP plots, regional distributions) were regenerated by Preflight Analysis and Conversion Engine (PACE) digital diagnostic tool to improve clarity and align with revised terminology.
- **Ethics Statement:** Moved exclusively to the Methods section.
- **Language Editing:** Professionally edited by with Grammarly tracked changes and clean copies are uploaded.
