# Supplementary material for: Understanding the determinants of treated bed net use in Ethiopia: A machine learning classification approach using PMA Ethiopia 2023 survey data
Source: PLoS One. 2025 Jul 7;20(7):e0327800. doi: 10.1371/journal.pone.0327800 (PMC12233236; doi:10.1371/journal.pone.0327800)
Supplement: Attachment — Submitted filename: Response_to_Reviewers_auresp_2.docx [file pone.0327800.s002.docx]

May 29, 2025

To: Editors of PLOS ONE

Response to reviewers’ comments

We want to thank you for inviting us to resubmit our manuscript entitled **“Understanding the Determinants of Treated Bed Net Use in Ethiopia: A Machine Learning Classification Approach Using PMA Ethiopia 2023 Survey Data”**. We are grateful to the reviewers for their time and effort in providing us with their valuable comments on our manuscript. We appreciate the comments the reviewers have given towards the improvement of this manuscript. We have noted all the comments and tried to address them in the revised manuscript accordingly. Moreover, we have thoroughly revised grammatical correctness across this manuscript and incorporated all the suggestions the reviewers have given. Point-by-point responses to the reviewer’s concerns are indicated below.

**Journal Requirements**

Please review your reference list to ensure that it is complete and correct. If you have cited papers that have been retracted, please include the rationale for doing so in the manuscript text, or remove these references and replace them with relevant current references. Any changes to the reference list should be mentioned in the rebuttal letter that accompanies your revised manuscript. If you need to cite a retracted article, indicate the article’s retracted status in the References list and also include a citation and full reference for the retraction notice.

Thank you for your reminder regarding the integrity and accuracy of the reference list. I have carefully reviewed all the references cited in my manuscript and confirm that none of them have been retracted to the best of my knowledge. Therefore, no retracted articles are cited, and no changes were necessary in the reference list related to retracted publications. I remain committed to maintaining the highest standards of scholarly integrity and appreciate your attention to this important matter.

**Reviewer Comments:**

**Reviewer #1**: Thank you for revising the manuscript based on comments. However, I still have some concerns. In general, I think you just replaced the term ownership by utilization. All the model comparison parameters are equal in both versions, which may not be true if you use different outcome variables.
1. Fig 3: There is no SNNP region. I think you are referring to South Ethiopia region

Thank you for your observation. You are correct the region referred to as "SNNP" in our figure stands for the Southern Nations, Nationalities, and Peoples' Region (SNNPR), which is how it is labeled in the original dataset. We acknowledge that this may have caused some ambiguity, especially given recent administrative changes. To address this, we have now included "SNNP" under the abbreviations and acronyms section of our manuscript for clarity. We appreciate your feedback.
2. Fig 4: Are these regions or households? How do you classify regions into quantiles? If they are households, put them in quantile order and then let’s see difference in graph.

Thanks for your comment on Figure 4.

In order to make it explicit, the x-axis in Figure 4 is not representing geographical regions, but quintiles of wealth. Quintiles are typically created by ordering all the families in the sample by some measure of their wealth (for example, income, assets) and then dividing them up into five groups of equal size, from "Lowest quintile" to "Highest quintile" which is already made by the data collectors.

Therefore, x-axis categories ("1. Lowest quintile", "2. Lower quintile", "3. Middle quintile", "4. Higher quintile", "5. Highest quintile") represent household groups ordered by their wealth.

As for your proposal to rank them by quantile and check for a difference, the figure presented is already ranked by wealth quintile, from lowest (3. Middle quintile) to highest (5. Highest quintile). This permits us to check the pattern of treated bednet use over these wealth groups.

3. Page 7: “The outcome variable, ITN use, was determined based on the question: 'Does your household have at least one insecticide-treated net?' with responses categorized as 'Yes' (1) or 'No' (0).” This is technically wrong. This measures “ownership of at least one ITN”, not use.
Please refer to this reference and see indicators and how they are measured.
<https://www.malariasurveys.org/documents/Household%20Survey%20Indicators%20for%20Malaria%20Control_FINAL.pdf>

Thank you for your thoughtful and important feedback. You are right to highlight the distinction between ITN ownership and ITN use. We sincerely apologize for the confusion caused by the wording in the manuscript. We have revised the manuscript to ensure that this distinction is clearly and consistently communicated. We have also referred to the recommended malaria survey indicators document you kindly shared to align our terminology with standard definitions. We greatly appreciate your guidance in improving the accuracy and clarity of our work.Our outcome variable is now written as under Variables Page 9 “The outcome variable is Treated bed net use (treated_ bed net): Binary indicator for household utilization of an insecticide-treated net (ITN) at the household level. In this study, insecticide-treated net (ITN) use was evaluated at the household level through two criteria: (1) the confirmed presence of at least one ITN during physical inspection, and (2) verification that the net was both insecticide-treated and actively deployed to assess the previous night condition during data collection (i.e., hung over a sleeping space) at the time of the survey, Even if there is a bednet which is not treated and not in the sleeping space it is recorded as ‘’No’’. ”
4. If you have number of family numbers and available ITN, why don’t you calculate ITN universal coverage?

Thank you for raising this important point. We agree that universal coverage (i.e., the proportion of the population with access to an ITN) is a valuable metric. In our analysis, we reported **household-level coverage** (42.6%, Fig. 1) because our study design and survey tools were structured to assess whether households owned and use at least one ITN, aligning with the WHO’s definition for household access.

As noted in the Methods section, we did not capture individual adherence (e.g., consistent use by all members), nor did we get granular data collected on intra-household ITN distribution beyond their usage. Thus, while we have the total number of household members and total ITNs available, we lack the necessary data on **which specific individuals had access to an ITN** (a requirement for calculating universal coverage).

**Reviewer #2**: If I have got it right, figure 2 provides number of ITNs (freq) on the Y-axis, and proportion as percent of total is given. I am wondering if this is the right way to show distribution of ITNs. Can the absolute numbers (# of ITNs per region) be transformed to show distribution per capita (household). This needs data on the total number of households in each region.

Thank you for your insightful observation. I hope you are referring to Figure 3 (bar chart of distribution of regions included). You are correct that Figure 3 provides the absolute quantity of what we can infer to be treated bednets (ITNs) on the y-axis, and the proportion that each region represents of the total.

Your recommendation to translate these absolute numbers to show the distribution per household or per capita is an excellent one and would undoubtedly show another image of the availability and usage with respect to each area's population. This would mean being aware of each area's number of households or population.

The current visualization effectively conveys which regions have the highest absolute numbers of treated bednets not per capita (house hold). That being said, a per capita or per household view could reveal disparities in coverage as a function of the population size of each region.

Appreciate you raising this important point. It is valuable feedback for considering alternative ways to visualize this data but the available data and my intention is region based visualization on this occasion.

**Reviewer #3**: Thank you for your thorough and thoughtful revisions to the manuscript. I appreciate the clear and comprehensive responses to all reviewer comments. The updated version of the manuscript demonstrates a high level of rigor and clarity.

Welcome, and thank you so much for your kind comments. I'm really grateful for the criticisms, which enhanced the manuscript. I'm relieved to know that the changes met your expectations, and I thank you for the support throughout the reviewing process.
