# Supplementary material for: Understanding the determinants of treated bed net use in Ethiopia: A machine learning classification approach using PMA Ethiopia 2023 survey data
Source: PLoS One. 2025 Jul 7;20(7):e0327800. doi: 10.1371/journal.pone.0327800 (PMC12233236; doi:10.1371/journal.pone.0327800)
Supplement: Attachment — Submitted filename: New Comments.docx [file pone.0327800.s003.docx]

**Understanding the Determinants of Treated Bed Net Use in Ethiopia: A Machine**

**Learning Classification Approach Using PMA Ethiopia 2023 Survey Data**

The manuscript is improved. A few issues require clarifications, and some editorial matters need to be attended to.

**Comments/Queries**

1. There are two figures under the label “Figure 2” (VIF Score of Independent Variables; and Distribution of regions with treated bed nets). Figure numbers need to re-numbered.
2. The titles of figures 2 & 3 (Distribution of regions with treated bed nets, Distribution of wealth quintile with treated bed nets) are confusing. It seems there is a need to be re-word them as “Distribution of treated bed nets by regions”, “Distribution of treated bed nets with wealth quintile”.
3. The Y-axis of these figures (figure 2 & 3) are written as “Count”. I understand “Count” is about the absolute numbers of bed nets. If so, it needs to be stated as such. Nonetheless, my comment about converting the absolute numbers to **per capita (or per household)** has been ignored even though the author seemed to have agreed to the proposition.
4. In page 18, it is unclear why the author considers the lower utilization of treated bed nets in urban areas as “*paradoxical*”.
5. The study did not assess perception of communities about malaria susceptibility. Thus, the statement “*This geographic disparity underlines the need for region-specific strategies, such as increasing community engagement in low-coverage areas and addressing misconceptions about malaria susceptibility among urban populations*” is out of context.
6. The fact that mid-tier households can prioritize better prevention measures than those who face extreme poverty or those who enjoy richness is an interesting finding. However, the concept of complacency is a bit confusing. It is understandable that the risk level among the rich may be lower and hence the need for using bed nets routinely may not be given high importance. While the term complacency maybe attributed to enjoyment of economic status, it is unclear how (as mentioned in page 18) it may be associated with failure to prioritize bed net use among the well to do households.

**Editorial**

In page 16, it seems the sentence after figure 6 (quoted below here) is incomplete. The problem could be a word or a phrase missing at the beginning.

*“the confusion matrix for the Random Forest model on classifying people with and without treated bed nets, it, therefore, follows that the model correctly predicted 4124 people without bed nets and 3500 people with bed nets, while it has misclassified 605 people without bed nets as with bed nets (Fig. 7).”*
