# Supplementary material for: Understanding the determinants of treated bed net use in Ethiopia: A machine learning classification approach using PMA Ethiopia 2023 survey data
Source: PLoS One. 2025 Jul 7;20(7):e0327800. doi: 10.1371/journal.pone.0327800 (PMC12233236; doi:10.1371/journal.pone.0327800)
Supplement: Attachment — Submitted filename: Response_to_Reviewers_auresp_3.docx [file pone.0327800.s004.docx]

6/9/2025

To: Editors of PLOS ONE

Response to reviewers’ comments

We want to thank you for inviting us to resubmit our manuscript entitled **“Understanding the Determinants of Treated Bed Net Use in Ethiopia: A Machine Learning Classification Approach Using PMA Ethiopia 2023 Survey Data”**. We are grateful to the reviewers for their time and effort in providing us with their valuable comments on our manuscript. We appreciate the comments the reviewers have given towards the improvement of this manuscript. We have noted all the comments and tried to address them in the revised manuscript accordingly. Moreover, we have thoroughly revised grammatical correctness across this manuscript and incorporated all the suggestions the reviewers have given. Point-by-point responses to the reviewer’s concerns are indicated below.

**Journal Requirements**

Please review your reference list to ensure that it is complete and correct. If you have cited papers that have been retracted, please include the rationale for doing so in the manuscript text, or remove these references and replace them with relevant current references. Any changes to the reference list should be mentioned in the rebuttal letter that accompanies your revised manuscript. If you need to cite a retracted article, indicate the article’s retracted status in the References list and also include a citation and full reference for the retraction notice.

Thank you for your reminder regarding the integrity and accuracy of the reference list. I have carefully reviewed all the references cited in my manuscript and confirm that none of them have been retracted to the best of my knowledge. Therefore, no retracted articles are cited, and no changes were necessary in the reference list related to retracted publications. I remain committed to maintaining the highest standards of scholarly integrity and appreciate your attention to this important matter.

**Response to Reviewer #2 Comments**

Dear Reviewer #2,

I sincerely thank you for your thoughtful and constructive feedback. I appreciate the time and effort you have taken to review my manuscript and your acknowledgment that the paper has improved. Below, I provide point-by-point responses to your comments, including clarifications and the corresponding revisions made to the manuscript.

**Comment 1:** There are two figures under the label “Figure 2” (VIF Score of Independent Variables; and Distribution of regions with treated bed nets). Figure numbers need to re-numbered.

**Response:**
Thank you for pointing this out. I have corrected the figure numbering to ensure consistency and clarity throughout the manuscript and supporting information. The duplication has been resolved, and each figure now has a unique and sequential number.

**Comment 2:** The titles of figures 2 & 3 (Distribution of regions with treated bed nets, Distribution of wealth quintile with treated bed nets) are confusing. It seems there is a need to be re-word them as “Distribution of treated bed nets by regions”, “Distribution of treated bed nets with wealth quintile”.

**Response:**
Thank you for this valuable suggestion. The figure titles have been revised to:

- **Figure 3**: “Distribution of Treated Bed Nets by Region”
- **Figure 4**: “Distribution of Treated Bed Nets by Wealth Quintile”
  This change aligns with standard conventions and improves the interpretability of the figures.

**Comment 3:** The Y-axis of these figures (figure 2 & 3) are written as “Count”. I understand “Count” is about the absolute numbers of bed nets. If so, it needs to be stated as such. Nonetheless, my comment about converting the absolute numbers to per capita (or per household) has been ignored even though the author seemed to have agreed to the proposition.

**Response:**
Thank you for this helpful clarification. I have revised the Y-axis labels of Figures 3 and 4 to explicitly state that they represent the **“Number of Households Using Treated Bed Nets”** to improve clarity and accuracy. I acknowledge the value of presenting the data on a per capita or household basis and had intended to incorporate this. However, due to limitations in the dataset, specifically the absence of consistent data on the number of bed nets per household member, I was unable to accurately compute per capita metrics. This limitation has now been acknowledged in the revised manuscript, and I have noted it as an important consideration for future research.

**Comment 4:** In page 18, it is unclear why the author considers the lower utilization of treated bed nets in urban areas as “paradoxical”.

**Response:**
Thank you for highlighting this ambiguity. I have rephrased the discussion to clarify that the term “paradoxical” was used to contrast the common assumption that urban areas, having better access to healthcare infrastructure, would logically show higher ITN usage. However, lower perceived malaria risk and behavioral complacency may reduce utilization. This clarification has been added to page 18 of the revised manuscript.

**Comment 5:** The study did not assess perception of communities about malaria susceptibility. Thus, the statement “This geographic disparity underlines the need for region-specific strategies, such as increasing community engagement in low-coverage areas and addressing misconceptions about malaria susceptibility among urban populations” is out of context.

**Response:**
You are correct, and I appreciate your careful reading. The statement has been revised to remove the reference to “misconceptions about malaria susceptibility” since perception data were not directly assessed. The revised sentence now focuses on strengthening community engagement in urban areas without making unsupported inferences.

**Comment 6:** The fact that mid-tier households can prioritize better prevention measures than those who face extreme poverty or those who enjoy richness is an interesting finding. However, the concept of complacency is a bit confusing. It is understandable that the risk level among the rich may be lower and hence the need for using bed nets routinely may not be given high importance. While the term complacency maybe attributed to enjoyment of economic status, it is unclear how (as mentioned in page 18) it may be associated with failure to prioritize bed net use among the well to do households.

**Response:**
Thank you for this insightful comment. I have revised the text to more carefully describe the observed phenomenon. Rather than attributing lower ITN use among wealthier households to “complacency,” I now discuss the possibility of a lower perceived malaria risk due to better housing or alternative prevention measures. The term “complacency” has been removed to avoid unintended implications and improve clarity. And can be found on page 19 on the revised manuscript as

*“This runs counter to the traditional narrative of linear wealth gradients in health access and suggests that mid-tier households may be more proactive in utilizing prevention measures. In contrast, lower ITN utilization among wealthier households might reflect a reduced perceived vulnerability to malaria, potentially due to better housing conditions or reliance on alternative protective measures, rather than a lack of concern or prioritization.”*

**Comment 7:** Editorial
In page 16, it seems the sentence after figure 6 (quoted below here) is incomplete. The problem could be a word or a phrase missing at the beginning.
“the confusion matrix for the Random Forest model on classifying people with and without treated bed nets, it, therefore, follows that the model correctly predicted 4124 people without bed nets and 3500 people with bed nets, while it has misclassified 605 people without bed nets as with bed nets (Fig. 7).”

Response: Thank you for pointing this out. You are right, there was a missing phrase at the beginning of the sentence, which made it unclear. I have revised the sentence for clarity and completeness.

Once again, I am truly grateful for your thoughtful critique, which has helped improve the rigor and clarity of the manuscript. I hope that the revised version addresses your concerns adequately.

Warm regards,
**Abraham Keffale Mengistu**
Email: [abreham_keffale@dmu.edu.et](mailto:abreham_keffale@dmu.edu.et)

Corresponding Author
